# Cardiovascular Risk Profiles amongst Women in a Multiethnic Population in Inner City Britain: A Potential Impact of Anaemia

**DOI:** 10.1155/2013/303859

**Published:** 2013-02-19

**Authors:** Julia Chackathayil, Jeetesh V. Patel, Paramjit S. Gill, Rahul Potluri, Ammar Natalwala, Hardeep Uppal, Deepthi Lavu, Reinhard Heun, Elizabeth A. Hughes, Gregory Y. H. Lip

**Affiliations:** ^1^University of Birmingham, Centre for Cardiovascular Sciences, City Hospital, Birmingham B18 7QH and Sandwell Medical Research Unit, Sandwell General Hospital, West Bromwich, West Midlands B71 4HJ, UK; ^2^Primary Care Clinical Sciences, University of Birmingham, Edgbaston, Birmingham B15 2TT, UK; ^3^Department of Cardiovascular Medicine, University of Manchester, Manchester M13 9NT, UK; ^4^Division of Medical Sciences, University of Birmingham, Edgbaston, Birmingham B15 2TT, UK; ^5^Department of Obstetrics and Gynaecology, University Hospital of North Staffordshire, Stoke-on-Trent, UK; ^6^Department of Psychiatry, Derby City General Hospital, Uttoxeter Road, Derby DE22 3NE, UK

## Abstract

The risk of diabetes is markedly reduced in men with iron deficiency anaemia (IDA). The nature of this relationship in women is not clear, nor is there information about the influence of ethnicity, given the increased susceptibility of diabetes amongst South Asians and Afro-Caribbeans. We reviewed 3563 patients with a diagnosis of anaemia from 2000 to 2007. The age-adjusted prevalence of vitamin B12 deficiency and IDA was calculated, together with cardiovascular comorbidities amongst Caucasians, South Asians, and Afro-Caribbeans. The prevalence of vitamin B12 deficiency (women only) or IDA was markedly higher in South Asians compared to Caucasians and Afro-Caribbeans. Among women with IDA, diabetes was more prevalent among South Asians (45%, 95% CI 39.0–51.0) compared to Caucasians (3.0%, 2.1–4.0); *P* < 0.001. Among South Asian women with vitamin B12 deficiency, the prevalence of diabetes was reduced 8.5% (5.2–12.0). South Asian women with vitamin B12 deficiency had a higher prevalence of myocardial infarction (MI) and ischemic heart disease (IHD), but this relationship was reversed in IDA. IDA is associated with a greater prevalence of diabetes in South Asian women, but it is not coordinated by a greater risk of macrovascular complications. Given the cardiovascular impact of diabetes in South Asians, this association merits further study in relation to its pathophysiological implication.

## 1. Introduction

Anaemia is an established risk factor for cardiovascular disease (CVD) outcomes in the general population [1], patients with chronic kidney failure, diabetes [2], acute coronary syndromes [3], and heart failure [4]. In the UK, the burden of CVD is more prominent amongst the country's ethnic minorities [5], and this is mainly due to the preponderance of CVD risk factors, such as diabetes mellitus and hypertension, which are most evident amongst South Asian and Afro-Caribbean ethnic groups [6, 7]. In addition, the CVD risk profiles amongst ethnic minorities living in the UK are likely to have deteriorated with migration from countries of origin to the UK, specifically from changes in dietary exposures [8, 9]. 

 A diet low in iron, folate, and vitamin B12 leading to nutrition-related anaemia could moderate CVD burden in this already high risk group [9, 10]. Vitamin B12 deficiency results in increased oxidative stress promoting a progression of atherosclerotic vessel disease [11]. On the contrary, iron deficiency anaemia (IDA) has been reported to confer a protective effect against atherosclerotic heart disease in premenopausal women [12]. Previous epidemiological studies have not explored the prevalence of anaemia and the distribution of CVD risk factors in a multiethnic population. 

The aim of the present study was to investigate the impact of anaemia on CVD risk profile among patients admitted to a multiethnic inner city teaching hospital in Birmingham,UK. We tested the null hypothesis that there were no significant differences in the effect of anaemia on CVD risk profile over a 7-year period (2000–2007). A database was compiled using data from national mortality registries and hospital records relating to the Sandwell and West Birmingham area, UK.

## 2. Research Design and Methods 

This was a retrospective, cross-sectional analysis of hospital cases. Information from the local West Midlands Regional Health Authority computerized Hospital Activity Analysis register was used to compile a database of all patients admitted to a large multiethnic inner city hospital during the period 2000–2007 (International Classification of Disease (ICD) 10th revision, codes 430–438). The coding system has been proven to be free of errors for definitive diagnoses [13]. The data were cross-referenced with the computerized Hospital Activity Analysis register, and furthermore, hospital case notes were reviewed for detailed information on demography, type of anaemia, and CVD risk factors (hypertension, diabetes mellitus, atrial fibrillation, myocardial infarction, ischemic heart disease, and hyperlipidaemia) to minimize any inaccuracies in coding.

### 2.1. Diagnostic Criteria and Definitions Used for Coding

Diagnosis was based on routine hospital practice which followed standard national guidelines [14]. A senior physician examined each patient admitted with symptoms and signs of CVD before performing any routine blood tests and further investigation. Diagnosis of anaemia was defined by hemoglobin levels <11.5 g/dL in females and <13.5 g/dL in males. IDA was defined as a Mean Corpuscular Volume (MCV) of less than 80fL along with a decreased total iron binding capacity saturation (<30%) and/or low serum iron (<11 umol/L) and/or serum ferritin (<20 ng/mL) and/or transferrin saturation of <12%. B12 deficiency anaemia was defined as MCV > 100 fL and vitamin B12 < 211 pg/mL. Anaemia of chronic disease was defined as anaemia in the absence of any other biochemical evidence of IDA or vitamin B12 deficiency in the presence of a chronic illness such as rheumatoid arthritis or cancer, for example. Blood pressure level >140/90 mmHg on at least two separate readings was indicative of hypertension. Diagnosis of diabetes was confirmed following patient's clinical history and biochemical evidence of at least two measurements of fasting blood sugar readings >7.0 mmol/l. A total cholesterol level >5.2 mmol/l was indicative of hyperlipidaemia. Diagnosis of atrial fibrillation was based on relevant clinical evidence supported by a 12-lead ECG. History of myocardial infarction was confirmed by ECG evidence, further supported by elevated levels of cardiac troponin. For the diagnosis of valvular heart disease, echocardiographic evidence for diagnosis of valve lesions was considered mandatory.

### 2.2. Data Analysis

We tested the null hypothesis that there were no significant differences in the effect of anaemia on CVD risk profiles over a 7-year period (2000–2007). The sample size was calculated for a power of 90% and an alpha error of 0.05, where a sample size of 115 in each group would be required to detect a 10% difference in the prevalence of anaemia in subjects with CVD risk profiles. 

Data were analyzed using SPSS version v14 (SPSS Inc., Chicago, IL, USA). Chi-square (*χ* ^2^) test was used to compare the categorical variables (e.g., differences in the prevalence of anaemia and CVD risk factors amongst the ethnic minorities). The prevalence of CVD risk factors was age and gender adjusted using the age distribution of total hospital admissions (2000–2007). 95% CI was calculated around means/proportions. 

## 3. Results

Between 2000 and 2007, there were 3563 hospital admissions, all diagnosed with anaemia, with complete clinical data available. Of all the patients with anaemia, 59% were women, 82% White, 13% South Asian, and 6% African Caribbean. Of these patients, 515 (14.5%) had IDA, 153 (4.3%) had vitamin B12 deficiency, 2534 (71.1%) presented with anaemia of chronic disease, and 361 (10.1%) were unspecified. Age-adjusted percentage of iron deficiency anaemia was significantly higherin South Asian women and men compared toWhiteand African Caribbean ethnic groups (*P* < 0.001; Table 1). Vitamin B12 deficiency anaemia was more common in South Asian women compared to Whitewomen (8.2% (95% CI 4.9–11.5) and 5.5% (4.4–6.6); *P* = 0.005), respectively. Anaemia of chronic disease was significantly more common in European White groups (73.0% (71.0–75.0); *P* = 0.001; Table 1). Across the study cohort, 17 (0.5%) patients had thalassemia and 113 (3.2%) had sickle cell anaemia. 

### 3.1. Risk Factors

Diabetes mellitus and ischemic heart disease were the most commonly encountered comorbidities at 8.2% and 10.1%. South Asian women had the highest prevalence of diabetes (*P* < 0.001). Ischemic heart disease was more prevalent amongst White and South Asian groups (*P* < 0.001) compared to Afro-Caribbeans. Whites had a higher prevalence of atrial fibrillation compared to Afro Caribbeans and South Asians (*P* < 0.001; Table 1). 

### 3.2. Anaemias and Cardiovascular Risk Factors

Among female patients with IDA, diabetes mellitus was significantly more prevalent among South Asian women (45% (39.0–51.0)) compared to White women (3.0% (2.1–4.0); *P* < 0.001). Among South Asian women with vitamin B12 deficiency anaemia, the prevalence of diabetes mellitus was (8.5% (5.2–12.0)). South Asian women with vitamin B12 deficiency anaemia showed a higher prevalence of myocardial infarction (4.3% (2.0–7.0)) and ischemic heart disease (26.6% (21.3–31.9)). South Asian women with IDA showed a low prevalence of myocardial infarction and no ischemic heart disease. Amongst White women, this relationship was not seen. Amongst women with IDA, the prevalence of hypertension was (28.0% (22.4–33.2)) and (21.0% (19.0-23.0)) in South Asians and Whites, respectively. Amongst women with vitamin B12 deficiency anaemia, the prevalence of hypertension was comparable in South Asians and Whites. Across the whole cohort, patients with anaemia of chronic disease showed higher prevalence of type 2 diabetes (*P* = 0.002), atrial fibrillation (*P* = 0.005), and ischemic heart disease (*P* = 0.065) irrespective of ethnicity (Table 2).

## 4. Discussion

The findings of this study suggest that among South Asian women with IDA, the prevalence of diabetes mellitus is exceptionally raised. This was not accompanied by an increased prevalence of ischemic heart disease—indeed, a paradoxical relationship is seen. The implication is that the presence of IDA in South Asian women may alleviate the risk of cardiovascular disease in a population that is susceptible to diabetes [7]. Further understanding into the pathophysiology of this relationship is required given the disturbing threat of ischemic heart disease on the Indian subcontinent [15]. 

 Prior, community-based studies among South Asian Indian women have demonstrated the prevalence of IDA and vitamin B12 deficiency anaemia mainly related to nutritional status [9]. The higher prevalence of IDA among women of South Asian origin living in Britain is likely to be explained by their life styles, including religious and cultural confines on certain foods [16]. 

 Vitamin B12 deficiency anaemia is considered a major health issue in Asia especially among vegetarians. Yajnik et al. [17] have reported the risk of vitamin B12 deficiency to be greater in vegetarians compared to nonvegetarians among South Asians. Vitamin B12 is largely found in food of animal origin and is a cofactor for methionine synthase which recycles homocysteine (HCY) to methionine. Deficiency results in increased serum HCY levels, increased oxidative stress leading to increased risk of atherosclerotic vessel diseases. Levels of HCY are recognised to be increased in South Asians and are associated with an increased risk of ischaemic heart disease and associated complications [10]. The present study shows that South Asian women with vitamin B12 deficiency anaemia had a higher prevalence of myocardial infarction and ischemic heart disease which confirms that vitamin B12 deficiency confers additional cardiovascular burden in this ethnic group [9, 10]. 

 Prospective studies have supported the hypothesis that iron plays a role in the development of type 2 diabetes and found a positive association between dietary heme-iron intake mainly from red meat and the risk of type 2 diabetes  [18]. Iron is a potent metal and could convert inactive free radicals to highly active free radicals which could potentially interfere with insulin synthesis and secretion in the pancreas, leading initially to insulin resistance and later to reduced insulin secretion. 

 In our study, the prevalence of diabetes mellitus was higher among South Asian women with IDA compared to those who had vitamin B12 deficiency anaemia. Early studies have demonstrated that iron metabolism influences glycosylation of hemoglobin A_1c_ (HbA_1c_) in patients with IDA, and levels of HbA_1c_ levels in such patients can be misleading [19]. El-Agouza et al. [20] reported that the mean HbA_1c_ level reduced significantly from 6.15%  ± 0.62 to 5.25%  ± 0.45 after iron treatment. Thus, concluding IDA is a potential source for misdiagnosis of uncontrolled diabetes mellitus among iron-deficient patients. Haemoglobinopathies, mainly conditions such as thalassemias and sickle cell anaemia, also contribute to false HbA_1c_ determinations in diabetic patients [21]. In South Asian women, both IDA and thalassemia should be considered before interpreting HbA_1c_ levels for the diagnosis of diabetes mellitus, but further work is warranted. 

 South Asian women with IDA also showed a higher prevalence of hypertension along with diabetes mellitus. Reduced hemoglobin levels suggest an increase in cardiac output secondary to a higher myocardial oxygen demand which can lead to mild-to-moderate hypertension [1]. Similarly, South Asian and White women with vitamin B12 deficiency showed higher prevalence of hypertension which could be attributed to the adverse effect of increased HCY levels [10]. 

 In the present study, South Asian women with IDA showed lower prevalence of myocardial infarction and no cases of ischemic heart disease at all. Among premenopausal women, the reduced risk of CVD is attributed to the female sex hormones [22]. However, IDA is considered a health problem due to menstrual loss. The protective effect against atherosclerotic heart disease among iron-deficient patients could be explained by lipid metabolism where excess iron acts as a catalyst for the modification of low density lipoprotein (LDL) leading to the progression of atherosclerotic plaques [23]. This study cohort of anemic patients showed less incidence of hyperlipidemia. Our results are consistent to Özdemir et al.[12], thus indicating a lower lipid profile in iron-deficient patients' hence, low risk of atherosclerotic diseases. 

### 4.1. Study Limitations

This is an observational study, and the cohort consisted of anemic patients who were admitted to an inner city hospital (2000–2007). Hence, the cause or effect of anaemia cannot be proved. The diagnosis of IDA, vitamin B12 deficiency anaemia, and anaemia of chronic disease was done at a single point of time; so, duration of anaemia is not known. The prevalence of vitamin B12 deficiency anaemia among South Asian women may suggest that most of them were vegetarians, but unfortunately, in this study diet, status was not recorded. Furthermore, data on pre-or postmenopause or medications such as metformin, iron supplements, or erythropoietin which could potentially change the anaemia or cardiovascular risk profile status were not included in the analysis. In addition, the impact of *β* thalassemia and sickle cell anaemia on HbA_1c_ measurements is obvious, but we do not know the influence of trait. One possible explanation for the higher prevalence of diabetes mellitus among South Asian women with IDA could be treatment with ferrous sulfate. Finally, no data were included on inflammatory cytokine levels which could have explained the higher prevalence of anaemia of chronic disease in this study cohort among Whites. 

## 5. Conclusions

South Asian women with IDA showed a higher prevalence of diabetes mellitus. Given the cardiovascular impact of diabetes in South Asians, this association merits further study in relation to its pathophysiological implication. Further work investigating IDA, underlying hemoglobinopathies and glycaemic control in this group, is needed. Vitamin B12 deficiency anaemia still remains a major health issue among South Asian women which needs to be tackled in order to provide a better healthcare for the management of CVD in this ethnic group. 

## Figures and Tables

**Table 1:** Prevalence of anaemia and cardiovascular risk profiles by ethnic group for the study cohort.

	Female (*n* = 2089)			Male (*n* = 1474)			*P* value*
	Caucasian (*n* = 1705)	South Asian (*n* = 266)	Afro-Caribbean (*n* = 118)	Caucasian (*n* = 1257)	South Asian (*n* = 117)	Afro-Caribbean (*n* = 100)
Age-adjusted percentage 3 types of anaemia, % (95% CI)							
Iron deficiency anaemia	15.2 (14–17)	22.0 (17.0–27.0)	10.0 (4.2–15.0)	12.1 (10.3–14)	20.0 (13.0–27.2)	4.0 (0.0–7.3)	<0.001
Vitamin B12 deficiency anaemia	5.5 (4.4–6.6)	8.2 (4.9–11.5)	0.8 (0–2.4)	3.4 (2.4–4.4)	0.7 (0–2.3)	0.0	0.005
Anaemia of chronic disease	73.0 (71.0–75.0)	65.2 (60.0–71.0)	53.0 (44.0–62.0)	73.0 (71.0–75.4)	70.5 (62.2–79.0)	62.0 (52.3–71.4)	0.001

Age-adjusted percentage of risk factors, % (95% CI)							
Type 2 diabetes	6.3 (5.1–7.4)	20.5 (16.0–25.4)	9.2 (4.0–14.4)	8.0 (6.2–9.1)	13.0 (7.0–19.1)	17.0 (9.4–24.0)	<0.001
Ischemic heart disease	9.2 (8.0–11.0)	11.4 (8.0–15.2)	3.5 (0.2–7.0)	11.0 (9.0–12.3)	15.0 (8.2–21.0)	0.0	<0.001
Myocardial infarction	1.6 (1.0–2.2)	2.0 (0.2–3.4)	0.0	3.0 (2.0–3.4)	2.4 (0–5.1)	0.0	0.094
Hypertension	15.3 (14.0–17.0)	29.2 (24.0–35.0)	25.0 (17.0–32.2)	13.0 (11.0–14.4)	0.0	23.4 (15.1–31.8)	0.996
Atrial fibrillation	6.6 (5.4–7.8)	1.1 (0–2.3)	3.5 (0.2–6.9)	5.0 (4.0–6.0)	0.6 (0–20)	2.4 (0–5.4)	<0.001

CI: confidence interval.

*On the basis of chi-square test across all groups.

Data are age-adjusted percent (95% CI).

**Table 2:** Impact of anaemia on the distribution of CVD risk profiles in women by ethnic group.

Risk factor	Type of anaemia	Caucasian (*n* = 1705)	South Asian (*n* = 266)	Afro-Caribbean (*n* = 118)	*P* value*
Type 2 diabetes	All anaemia	6.3 (5.1–7.4)	20.5 (16.0–25.4)	9.2 (4.0–14.4)	<0.001
	Iron deficiency anaemia	3.0 (2.1–4.0)	45.0 (39.0–51.0)	0.0	<0.001
	Vitamin B12 deficiency anaemia	10.5 (9.1–12.0)	8.5 (5.2–12.0)	0.0	0.756
	Anaemia of chronic disease	7.0 (5.3–8.0)	15.2 (11.0–20.0)	10.1 (5.0–16.0)	0.002

Ischemic heart disease	All anaemia	9.2 (8.0–11.0)	11.4 (8.0–15.2)	3.5 (0.2–7.0)	0.005
	Iron deficiency anaemia	12.0 (10.2–13.3)	0.0	0.0	0.206
	Vitamin B12 deficiency anaemia	12.4 (10.8–13.9)	26.6 (21.3–31.9)	0.0	0.168
	Anaemia of chronic disease	9.0 (7.2–10.0)	8.4 (5.1–12.0)	0.0	0.065

Myocardial infarction	All anaemia	1.6 (1.0–2.2)	2.0 (0.2–3.4)	0.0	0.356
	Iron deficiency anaemia	2.3 (1.6–3.0)	2.4 (0.5–4.2)	0.0	0.731
	Vitamin B12 deficiency anaemia	1.0 (0.5–1.4)	4.3 (2.0–7.0)	0.0	0.171
	Anaemia of chronic disease	1.6 (1.0–2.2)	2.0 (0.3–4.0)	0.0	0.632

Hypertension	All anaemia	15.3 (14.0–17.0)	29.2 (24.0–35.0)	25.0 (17.0–32.2)	0.918
	Iron deficiency anaemia	21.0 (19.0–23.0)	28.0 (22.4–33.2)	0.0	0.786
	Vitamin B12 deficiency anaemia	24.0 (22.0–26.0)	23.4 (18.3–28.5)	0.0	0.601
	Anaemia of chronic disease	13.4 (12.0–15.0)	23.0 (18.0–28.0)	19.4 (12.3–27.0)	0.970

Atrial fibrillation	All anaemia	6.6 (5.4–7.8)	1.1 (0–2.3)	3.5 (0.2–6.9)	<0.001
	Iron deficiency anaemia	10.3 (9.0–12.0)	2.4 (0.5–4.2)	0.0	0.024
	Vitamin B12 deficiency anaemia	8.8 (7.4–10.1)	0.0	0.0	0.583
	Anaemia of chronic disease	6.1 (0.0–2.1)	1.0 (0.0–2.1)	7.1 (3.0–12.0)	0.005

Data are age-adjusted percent (95% CI).

*On the basis of chi-square test across all groups.
